# Supplementary material for: Healthcare utilization among pre-frail and frail Puerto Ricans
Source: PLoS One. 2023 Jan 12;18(1):e0280128. doi: 10.1371/journal.pone.0280128 (PMC9836292; doi:10.1371/journal.pone.0280128)
Supplement: S1 Table — (DOCX) [file pone.0280128.s001.docx]

| **S1 Table. Frailty Criteria Characteristics at Baseline (2002-2003) and Follow-up (2006-2007).** | | | | | |
| --- | --- | --- | --- | --- | --- |
| **Baseline (n=3,040)** | | | **Follow-up (n=2,121)** | | |
| **Criteria** | **N (%)** | **Missing frailty items** | **Criteria** | **N (%)** | **Missing frailty items** |
| Shrinking (weight loss) |  |  | Shrinking (weight loss) |  |  |
| Yes | 661 (21.7) |  | Yes | 451 (21.3) |  |
| No | 2,379 (78.3) |  | No | 1,670 (78.7) |  |
| Weakness (leg stand) |  |  | Weakness (leg stand) |  |  |
| Yes | 1,765 (58.1) |  | Yes | 1,290 (60.8) |  |
| No | 1,275 (41.9) |  | No | 831 (39.2) |  |
| Full of energy |  |  | Full of energy |  |  |
| Yes | 2,499 (82.2) |  | Yes | 1,746 (76.7) |  |
| No | 541 (17.8) |  | No | 375 (17.7) |  |
| Slowness (timed up and go) |  | 108 | Slowness (timed up and go) |  | 15 |
| <20^th^ percentile | 2,194 (74.8) |  | <20^th^ percentile | 1,615 (76.7) |  |
| >20^th^ percentile | 738 (25.2) |  | >20^th^ percentile | 491 (23.3) |  |
| Physical activity |  |  | Physical activity |  |  |
| Yes | 1,352 (44.5) |  | Yes | 1,685 (79.4) |  |
| No | 1,688 (55.5) |  | No | 436 (20.6) |  |
| Follow-up data includes participants that completed baseline questionnaires at Wave 2 in 2007-2008. Participants were excluded if they were missing baseline criteria for covariates and baseline frailty criteria. Participants were additionally excluded if they were missing information on the main outcome of ER visits, hospital stays, and doctor visits. | | | | | |
